# Featured Prebiotic Agent: The Roles and Mechanisms of Direct and Indirect Prebiotic Activities of Lactoferrin and Its Application in Disease Control

**DOI:** 10.3390/nu15122759

**Published:** 2023-06-15

**Authors:** Zhen-Shu Liu, Po-Wen Chen

**Affiliations:** 1 Chronic Diseases and Health Promotion Research Center, Chang Gung University of Science and Technology, Chiayi 61363, Taiwan; zsliu@mail.mcut.edu.tw; 2 Department of Safety, Health and Environmental Engineering, Ming Chi University of Technology, New Taipei City 24301, Taiwan; 3 Department of Veterinary Medicine, National Chung Hsing University, Taichung 40249, Taiwan

**Keywords:** recombinant lactoferrin, lactoferricin, prebiotic, probiotic, antibacterial activity, lactobacilli, bifidobacteria

## Abstract

Lactoferrin (LF) is a glycoprotein found in mammalian milk, and lactoferricin is a peptide derived from LF hydrolysate. Both LF and lactoferricin (LFcin) have diverse functions that could benefit mammals. Bovine LF (BLF) and BLFcin exhibit a wide range of antimicrobial activities, but most probiotic strains are relatively resistant to their antibacterial effects. BLF and BLF hydrolysate can promote the growth of specific probiotics depending on the culture conditions, the dose of BLF or BLF-related peptides, and the probiotic strains used. BLF supplementation has been shown to modulate several central molecular pathways or genes in *Lacticaseibacillus rhamnosus* GG under cold conditions, which may explain the prebiotic roles of BLF. LF alone or in combination with selected probiotics can help control bacterial infections or metabolic disorders, both in animal studies and in human clinical trials. Various LF-expressing probiotics, including those expressing BLF, human LF, or porcine LF, have been developed to facilitate the combination of LFs with specific probiotics. Supplementation with LF-expressing probiotics has positive effects in animal studies. Interestingly, inactivated LF-expressing probiotics significantly improved diet-induced nonalcoholic fatty liver disease (NAFLD) in a mouse model. This review highlights the accumulated evidence supporting the use of LF in combination with selected LF-resistant probiotics or LF-expressing probiotics in the field.

## 1. Introduction

Lactoferrin (LF) is an 80 kDa glycoprotein that belongs to the family of transferrin proteins and functions as an iron-binding protein. It was first discovered in cow’s milk in 1939 [1], and in 1960, it was identified as the primary iron-binding protein in human milk [2]. Subsequently, LF has been found in most mucosal secretions, such as tears, saliva, vaginal mucus, seminal plasma, nasal and bronchial secretions, bile, gastrointestinal fluids, and urine [3]. Although LFs are present in relatively low concentrations in plasma, they are predominantly secreted by neutrophils during inflammation responses [4]. Up to now, LF has been identified in most mammals, including primates, carnivores, rodents, lagomorphs, artiodactyls, perissodactyls, Proboscidea, Didelphimorphia, and Cingulata [5]. The two most studied and well-characterized LFs are bovine LF (BLF) and human LF (HLF). Both BLF and HLF have been associated with pleiotropic activities such as anti-inflammatory, antimicrobial, antioxidant, and immune-regulating properties in various fields [6,7,8]. Therefore, BLF and HLF are thought to be promising candidates for the treatment of anemia, inflammation, microbial infections, cancer, and immunomodulatory activities [9,10,11,12].

The antibacterial activity of LF was the initial activity to be identified for LF, and both BLF and HLF have been documented to display wide-spectrum antimicrobial activity against various pathogens, including antibacterial, antiviral, antifungal, and antiparasitic activities [12,13,14,15]. As for the antibacterial activities of LF, HLF and BLF possess both bacteriostatic and bactericidal activities [14]. Their iron-sequestering property is the basis of the bacteriostatic effect, and this could help to deprive bacteria of free iron for pathogenic bacterial growth [16,17]. In addition, the bactericidal activity of LF is attributed to an *N*-terminal fragment of LF, lactoferricin (LFcin). This peptide was firstly purified from LF hydrolysate after the pepsin digestion of complete BLF and HLF. Importantly, studies have supported that bovine and human LFcins exhibit stronger bactericidal activity against a number of bacterial strains than native LFs [14,15,18].

The concept of prebiotics was initially introduced by Gibson and Roberfroid in the 1990s. They emphasized that prebiotics are nondigestible food ingredients that beneficially affect the host by selectively stimulating the growth and/or activity of a limited number of bacteria in the colon, thereby improving host health [19]. In December 2016, an updated definition was proposed during a panel of experts in microbiology, nutrition, and clinical research convened by the International Scientific Association of Probiotics and Prebiotics (ISAPP) in London, United Kingdom. According to this revised definition, a prebiotic refers to a substrate that is selectively utilized by host microorganisms, conferring a health benefit [20]. It is important to note that the term “substrate” refers to any substance used for growth through nourishment, which is selectively utilized and should have effects limited to specific bacterial communities. Therefore, purely antimicrobial agents should be excluded as prebiotics [20]. While carbohydrate-based products are the most well-known prebiotics, some noncarbohydrate compounds such as polyphenols, minerals, and polyunsaturated fatty acids have also been proposed as prebiotic candidates [20,21]. To date, prebiotic supplementation has been shown to exert a protective role on human health endpoints. For instance, these functional ingredients in foods influence the gut microbiota by stimulating the growth of beneficial microbes and the production of beneficial metabolites. This, in turn, directly benefits the host and provides protection against pathogens, while maintaining a balanced gut ecosystem [21,22,23].

Probiotics were originally defined as “live microbial feed supplements that beneficially affect the host, improving its intestinal microbial balance” [24]. However, this definition has since been revised, and probiotics are currently defined as “live microorganisms that, when administered in appropriate doses, confer health benefits to the host” [25]. Probiotics, and functional factors derived from probiotics, have been shown to play important roles in maintaining intestinal homeostasis in the host. They can also contribute to animal health, nutrition, growth, and production performance. Therefore, probiotic-related products have become some of the fastest-growing products in the food industry, as there is considerable scientific evidence for their positive health effects on both consumers and animals [26,27,28]. The relationship between LF and probiotics has also been investigated, and early studies suggest that BLF and HLF can exhibit prebiotic activities on several probiotic strains [29,30,31]. However, mixed results have been observed in different studies. Later studies further explored the effects of LF on various probiotic strains under different conditions. For example, the data of Chen et al. revealed that, although LF displays a wide spectrum of antibacterial activities against various bacterial pathogens, most tested probiotic strains are relatively resistant to the antibacterial effects of LF or LF-derived peptides [32,33]. Moreover, this group also reported for the first time that BLF can promote the growth of several probiotic strains whose growth was blocked in cold environments [33]. Notably, Liu et al. further explored the molecular pathways of BLF that could promote the growth of a specific probiotic strain [34]. Recently, the potential role of LF in promoting the growth of probiotics in the gut and reproductive tract has been summarized as well [35,36].

Recent findings have provided a detailed and updated elucidation of the molecular regulatory mechanism of LF on the growth of specific probiotics. Therefore, this review highlights recent discoveries and offers updated perspectives on the impact of LF on the growth of specific probiotics. We emphasize the potential roles of LF in positively regulating gut microbiota and discuss the benefits of combining LF with specific probiotics. Furthermore, we discuss various studies that have attempted to construct recombinant probiotic strains capable of expressing HLF, BLF, or porcine lactoferrin (PLF) to facilitate the combination of LFs with specific probiotics.

## 2. Contrasting Antibacterial Effects of Lactoferrins on Pathogens and Probiotics

To dissect the prebiotic ability of LF and LF-related peptides, it is necessary to first describe their antibacterial activities. Although LF is known to have various protective functions in the mammalian body [37,38,39,40], its antimicrobial activity has been the most studied feature [41]. The mechanisms of the antimicrobial activities of LF, LFcins, and other LF-derived peptides have been well elucidated and reviewed in some studies [5,42,43]. Although LF has been identified in many mammals, the antibacterial activities of LF or LF-derived peptides from bovines and humans have been the most studied to date [42]. For example, LF or LF-derived peptides from bovines and humans have been reported to display antibacterial activities against numerous Gram-positive bacteria, including *Bacillus*, *Bifidobacterium*, *Clostridium*, *Corynebacterium*, *Enterococcus*, *Lactobacillus*, *Listeria*, *Micrococcus*, *Staphylococcus*, and *Streptococcus* species. In addition, these LF-related proteins or peptides display antibacterial activities against numerous Gram-negative bacteria, including *Aggregatibacter*, *Bacteroides*, *Escherichia*, *Enterobacter*, *Campylobacter*, *Helicobacter*, *Legionella*, *Proteus*, *Pseudomonas*, *Salmonella*, *Klebsiella*, *Vibrio*, and *Yersinia* species [5,42,43]. However, it is worth noting that the antibacterial effects of LF on probiotic bacteria are often in contrast to its effects on pathogenic bacteria. Our recent studies have shown that, although LF displays a wide spectrum of antibacterial activities against various bacterial pathogens, most tested probiotic strains are relatively resistant to the antibacterial activities of LF or LF-derived peptides [32,33].

In summary, BLF, HLF, and their derived peptides exhibit inhibitory effects on the growth of most tested pathogenic bacteria. The antibacterial activity of LFs, on the other hand, can be tolerated by most probiotic strains at the same LF level. However, it is worth noting that the growth of various probiotic strains may still be inhibited by relatively higher concentrations of BLF or BLF hydrolysate (as summarized in Table 1). Functional foods combining LF and specific probiotics have been marketed; however, the added LF may impede the activities of certain probiotics. Nevertheless, LF has been found to promote the growth of several probiotics under specific environmental conditions and dosages. The conditions under which LF can promote the growth of probiotics are discussed and summarized in the following section.

## 3. The Potential Prebiotic Activity of Lactoferrin In Vitro

In an early study, BLF from mature milk increased the growth of *Bifidobacterium longum* subsp. *infantis* and *B. breve* in vitro in a dose-dependent manner, while much less growth-promotion activity was observed for *B. bifidum*. In contrast, HLF from mature milk promoted the growth of *B. bifidum* and was inactive for *B. infantis* and *B. breve*, while BLF from colostrum was devoid of bifidobacterial growth-promotion activity. Thus, the bifidogenic ability of BLF and HLF are dependent on bifidobacterial strains. This study also demonstrates that the ability of LF to promote the growth of *Bifidobacterium* spp. in vitro is independent of the iron saturation level for LF and suggests that the binding of LF to bifidobacterial cells may be partially involved, but is not sufficient for the stimulation of bifidobacterial growth [31]. Intriguingly, a report found that both LF and LFcin either enhance the growth of certain *bifidobacteria* and *lactobacilli* or they have no effect at all on their growth [47]. Another study supported the notion that low doses of BLF hydrolysate (0.01 to 1 mg/mL) can stimulate the growth of *B. infantis* (ATCC 15696 and 15697) and *B. breve* ATCC 15700 [45]. On the other hand, Chen et al. reported that BLF can still dose-dependently inhibit the growth of a series of probiotic strains; although, higher minimal inhibitory concentrations of BLF are required to completely inhibit the growth of most probiotics [46].

Collectively, the mixed effects of BLF on the growth of specific probiotics could be partially explained by inconsistent assay strategies, in which various concentrations, iron-saturated forms, and LF purities have been adapted [31,44,46]. Intriguingly, a recent report further demonstrated that BLF displayed consistent prebiotic activities on certain probiotics when these probiotic cells were cultured in a cold (stress) environment [33]. For example, BLF displayed inconsistent prebiotic activity on the 14 probiotics at 37 °C. However, in a 22 °C environment, the growth of *B. breve*, *Loigolactobacillus coryniformis* subsp. *coryniformis*, *L. delbrueckii*, *L. acidophilus*, *B. angulatum*, *B. catenulatum*, and *Lactiplantibacillus paraplantarum* were completely blocked, but these probiotics started regrowing in the presence of BLF (1–32 mg/mL) in a significant and dose-dependent manner. Accordingly, BLF also significantly increased the growth of *Pediococcus pentosaceus*, *L. rhamnosus*, and *Lacticaseibacillus paracasei* (when their growth was retarded by incubation at 22 °C). Therefore, BLF indeed conferred strong prebiotic activity in 10 probiotic strains at 22 °C [33]. Moreover, an early report tried to evaluate the activity of LF (the type of LF was not described) on the multiplication of probiotic *Lacticaseibacillus casei* in Minas fresh cheese, which was produced and stored at 5 °C over 28 days. This study observed that, when tested in vitro, *L. casei* multiplication was stimulated by LF at a concentration of 2 mg/mL, but this activity was not observed in the cheese when it was stored at 5 °C, even when lactoferrin was added at 4 mg/g [48]. To support this, in our unpublished data, we also found that BLF could not promote the growth of tested probiotics that were cultured at 4 °C [33]. While certain data mentioned above may not be directly applicable to the utilization of BLF for prevention or treatment, as the experiments were conducted under nonphysiological conditions (4 °C, 5 °C, 22 °C), they may still bear relevance to the incorporation of dairy products infused with probiotics and BLF. To substantiate this notion, Duran et al. investigated the effect of BLF on the microbiological properties of raw milk during cold chain storage. The data revealed that BLF had no inhibitory effect on lactic acid bacteria. However, BLF significantly inhibited the growth of *Pseudomonas* spp. and Coliform. The study suggests that BLF can be utilized as a natural antimicrobial agent in cold liquid food systems [49]. Furthermore, a recent review has summarized the potential of using LFcin-related peptides against food pathogens and suggests the utilization of LFcin for food preservation [50].

Regarding the prebiotic effects of BLF hydrolysate on probiotics, BLF hydrolysate at concentrations ranging from 0.5 to 128 mg/mL did not stimulate the growth of any tested probiotic strain, including *L. acidophilus* ATCC 4356, *Lacticaseibacillus rhamnosus* ATCC 7469, *Ligilactobacillus salivarius* ATCC 11741, *Limosilactobacillus reuteri* ATCC 23272, *L. rhamnosus* ATCC 53103, *L. acidophilus* BCRC 14065, *L. fermentum* ATCC 11739, *L. coryniformis* ATCC 25602, *B. infantis* ATCC 15697, *B. longum* ATCC 15707, *B. bifidum* ATCC 29521, *P. acidilactici* ATCC 8081, and *B. lactis* BCRC 17394 [32]. However, BLF hydrolysate inhibited the growth of *L. acidophilus* ATCC 4356, *L. salivarius* ATCC 11741, *L. rhamnosus* ATCC 53103, *B. longum* ATCC 15707, and *B. lactis* BCRC 17394 in a concentration-dependent manner, ranging from 1 to 32 mg/mL of BLF hydrolysate [32]. In contrast, Kim et al. reported that BLF hydrolysate at concentrations ranging from 0.01 to 1 mg/mL could promote the growth of three bifidobacterial strains, including *B. bifidum* ATCC 15696, *B. infantis* ATCC 15697, and *B. breve* ATCC 15700 [45]. To support this, a recent study has evaluated the effect of LF addition on the viability of *L. acidophilus* starter in the skimmed milk samples after fermentation, and the findings revealed that the levels of the viable counts of *L. acidophilus* in the stirred yogurt–LF sample were significantly increased in comparison with stirred yogurt alone and these results indicated that the existence of LF in fermented milk helped to improve the growth of the *L. acidophilus* starter due to the prebiotic effects of LF on enrichment fermented culture [51]. Nevertheless, the observed differences in the effects of BLF hydrolysate on the growth of the tested probiotics may be explained by the different concentrations of BLF hydrolysate tested, such as 0.01 to 1 mg/mL versus 0.5 to 128 mg/mL. In our previous unpublished data, we found that the growth of certain bacterial pathogens was occasionally enhanced by apo-BLF when the tested BLF concentration was below the minimum inhibitory concentration (MIC) values against these pathogens.

Since LF or LF hydrolysate can play various roles in vivo, we summarized only the potential prebiotic or inhibitory activity of BLF and BLF hydrolysate in vitro in Table 1. However, it should be noted that the majority of these works were completed during earlier years, and we conducted the majority of the work in the field. For example, we initially examined the prebiotic activities of BLF or BLF hydrolysates across multiple probiotic strains; further, we determined the effects of culture conditions, including anaerobic conditions, aerobic conditions, and different temperatures (37 °C, 28 °C, 22 °C, and 4 °C), which may affect the prebiotic activities of LF [32,33,46].

## 4. The Direct and Indirect Prebiotic Roles of Lactoferrin In Vivo: Benefits of LF Supplementation on Gut Microbiota or Disease Control

An early study reported that the supplementation of adapted formula with 100 mg/100 mL of BLF could establish a “bifidus flora” in half of the infants given the formula at three months of age [52]. Mastromarino et al. investigated the association between LF and beneficial microbiota in breast milk and infant feces, demonstrating that the LF concentration in the feces of 30-day-old term infants was significantly correlated with the maternal mature milk LF concentration (*p* = 0.030). The concentration of fecal bifidobacteria and lactobacilli was also associated with the concentration of fecal LF three days after delivery (*p* = 0.017 and *p* = 0.026, respectively), suggesting that high levels of fecal LF in neonates, particularly in the first days of life, could play an important role in the initiation or composition of the neonatal gut microbiota [53]. However, in our opinion, the correlation between the LF concentration in milk and that in fecal microflora may not reflect the direct prebiotic activities of LF in the gut due to the various factors present in human milk. Notably, Dix et al. recently investigated the bioavailability of microencapsulated BLF and its effect on the gut microbiome in a double-blind, randomized, cross-over trial [54]. The study observed that phylum-level changes in the microbial community profiling were detected post-supplementation in the second trial arm, particularly in those receiving microencapsulated BLF. However, the study did not dissect the changes in the probiotic populations. Nevertheless, these findings suggest that BLF supplementation may have beneficial effects on the microbiome.

Moreover, in very-low-birth-weight infants, two doses of recombinant HLF daily from days 1 to 28 of life were found to reduce *Enterobacter* and *Klebsiella* while increasing Citrobacter in the infants’ feces [55]. Additionally, an oral administration of BLFcin could efficiently maintain gut microbiota homeostasis in an enterohemorrhagic EHEC O157:H7 mouse model [56]. Importantly, a recent study also found that a 16-week intervention of LF might affect the gut microbiota profiles differently in young and middle-aged APP/P1 mice [57]. Furthermore, BLF was reported to positively modulate the gut microbiota and Toll-like receptors (TLRs) in mice with dysbiosis induced by antibiotics. However, the study did not investigate which specific probiotic population was enhanced by the BLF supplement [58]. Sun et al. reported that administering 100 mg/kg (body weight) of BLF orally for 12 weeks prevented obesity in mice with an obese condition induced by a high-fat diet. The study also found that BLF reduced gut inflammation and systemic LPS levels, indicating that BLF has a positive effect on the gut microbiota in obese mice [59]. A crucial finding of a review is that the available evidence suggests that an oral administration of BLF can decrease the occurrence of late-onset sepsis and necrotizing enterocolitis (NEC) stage II or greater in preterm infants without any negative effects, and this administration decreases the intestinal injury in experimental NEC by downregulating inflammation and upregulating cell proliferation [60,61,62]. Moreover, LF also holds promise as an agent against streptococcal infections, likely due to its ability to display antibacterial activities, modulate the host inflammatory response, and influence disease outcome [63]. Importantly, LF exhibits dual functionality, demonstrating both antibacterial and antibiofilm activities. For instance, a study observed an elevation in *Streptococcus mutans* biofilm formation in human breast milk samples obtained between 3–9 months postpartum. However, the addition of Lactoferrin at an average milk concentration of 3 mg/mL significantly reduced biofilm formation across all dilutions [64]. Similarly, HLF exhibits slightly stronger antibacterial activity compared to BLF against specific strains of *A. baumannii*. Additionally, both BLF and HLF have the ability to inhibit the formation of *A. baumannii* biofilms [65]. Finally, in vitro and in vivo assays have demonstrated the ability of BLF, HLF, and camel LF to effectively reduce the occurrence of infectious *E. coli* and *Acinetobacter baumannii* [66].

Regarding the potential roles of LF in the treatment of bacterial vaginosis, Otsuki et al. underscored the significance of oral and vaginal administration of LF in facilitating successful childbirth among women. Notably, after one month of lactoferrin therapy, the vaginal microbiomes of these women displayed a marked prevalence of *Lactobacillus* spp. This study postulates that the administration of LF to humans holds potential for averting refractory vaginitis, cervical inflammation, and preterm delivery [67]. To support this, Pino et al. also conducted a study to characterize the bacterial biota of women affected by bacterial vaginosis. The findings of this study demonstrated that both the 100 mg and 200 mg LF vaginal pessaries significantly reduced the occurrence of bacteria commonly associated with bacterial vaginosis, including *Gardnerella*, *Prevotella*, and *Lachnospira*. Additionally, these treatments notably increased the presence of *Lactobacillus* species. Notably, the balance of the bacterial biota was maintained for up to 2 weeks after treatment, but this effect was observed only in women treated with 200 mg lactoferrin pessaries [66]. Hence, the administration of LF has been shown to effectively enhance the in vivo growth of specific probiotic strains and exhibit efficacy against infectious pathogen infections.

On the other hand, LF supplementation has been shown to have beneficial effects on the management of obesity in both human and rodent studies [59,68,69,70,71]. Intriguingly, the combined intervention of hypoxia and LF improved the body weight, blood, and pathological indices in high-fat feeding mice by restoring gut microbiota composition and bile acid profile [72]. Moreover, a recent study evaluated the association of genetic variants in LF metabolism-related genes with the prevalence of metabolically healthy obesity and metabolically unhealthy obesity, and the low-density lipoprotein receptor-related protein (LRP) 2 rs2544390, LRP1 rs4759277, LRP1 rs1799986, LTF rs1126477, LTF rs2239692, and LTF rs1126478 were genotyped. This study has demonstrated that the selected LF and LF receptor-related gene variants may be associated with the prevalence of metabolically healthy or metabolically unhealthy obesity [73]. Sato et al. had investigated the efficacy of LF on fertility problems in overweight and obese mothers, and this study demonstrated that LF ingestion can improve fertility in overweight/obese females and suppress the health problems of their offspring [74]. However, dietary LF has been found to cause significant weight and fat loss, mostly under calorie-restricted conditions [75,76,77,78]. Collectively, the above studies support that LF supplements can be good candidates for treating infection diseases (pathogenic bacteria) and metabolic disorders in humans.

Overall, BLF is the most used form of LF in human and animal studies, with administration at varying dosages, formulations, and intervention periods. These studies have demonstrated the positive impact of LF on gut microbiota and disease control. However, there has been a lack of research on the in vivo effects of LF on specific probiotic populations in the gut. This is reasonable, given that gut microbiota play a crucial role in host health rather than individual probiotic strains, as highlighted in previous studies [79,80]. It is expected that this trend will continue.

Notably, a recent review has described the antimicrobial and prebiotic activities of LF in the female reproductive tract [36,36]. Some of our previous findings regarding the prebiotic potential of LF are discussed in that review. However, early studies have reported relatively low concentrations of LF in the vaginal and cervical secretions of healthy women, ranging from 0.16 to 154 μg/mL depending on the stage of the menstrual cycle [81,82]. Based on the in vitro assay data presented in Table 1, we consider that LF levels in the vaginal and cervical secretions of healthy women may not be sufficient to promote the growth of most probiotics. Nonetheless, LF supplements with probiotics have shown promise in controlling vaginal infections [36].

## 5. The Mechanism of LF Becoming a Prebiotic Agent

Previous studies have attempted to elucidate the prebiotic effects of LFs on specific probiotic strains. The prebiotic activity of LF or LFcin may be attributed, in part, to the physicochemical properties of the probiotic surface components [83,84,85,86]. Additionally, although certain LF-binding proteins have been identified in the membrane and cytosolic fractions of *Bifidobacterium longum* [29,87,88], the binding of LF to bifidobacterial cells alone is insufficient to stimulate bifidobacterial growth [31,35]. Notably, LF-binding proteins have been widely identified on the surface of various pathogenic bacteria [89,90,91,92,93,94]. However, to our knowledge, the existence of LF-binding proteins on lactobacilli has not been reported to date, and further investigation is necessary to explore this topic. As shown in Table 1, given that LF, LFcin, and LF hydrolysate have all been shown to exhibit prebiotic activity on certain strains of probiotics, we propose that LF-binding proteins may play a minor role in their prebiotic effects.

**Figure 1:**
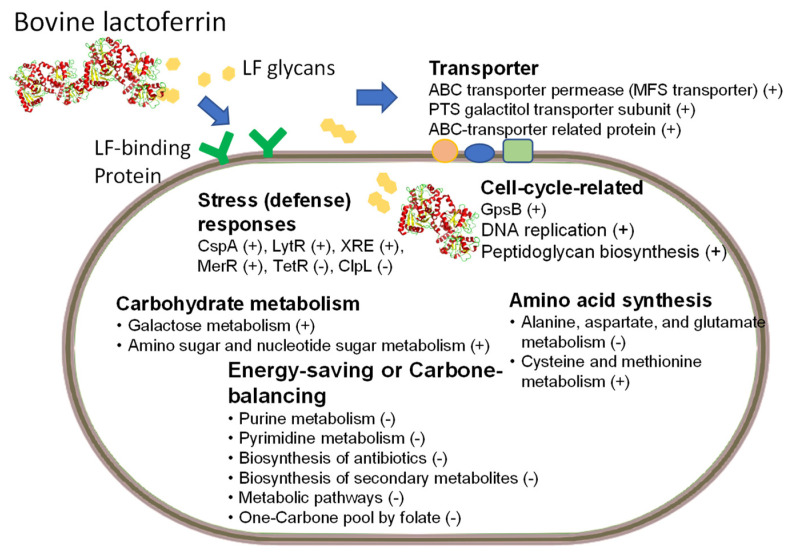
Featured prebiotic ability of BLF: *Lacticaseibacillus rhamnosus* GG strain was cultured in MRS medium with and without various concentrations of bovine lactoferrin (BLF) at 22 °C. Central molecular pathways or genes are shown that were modulated by bovine lactoferrin (LF) supplementation in LGG incubated in a cold environment (22 °C). Increased or decreased expression is described by a plus symbol, “+”, or a minus symbol, “-”, respectively. The contents of the figure have been modified and combined from two recent reports [34,35]. CspA: a cold-shock protein; LytR: LytR-family transcriptional regulator; XRE: XRE-family transcriptional regulator; MerR: MerR-family transcriptional regulator (activator of bmr gene); TetR: TetR-family transcriptional regulator; Clp: ATP-dependent Clp protease ATP-binding protein; GpsB: cell division protein.

Chen et al. investigated the ability of BLF to enhance the growth of *L. rhamnosus* GG (LGG) when the strain’s growth was impeded by cold conditions (22 °C). To elucidate the underlying molecular mechanisms, this group employed a transcriptome analysis. This study revealed, for the first time, that BLF can modulate several central pathways to enhance the growth of LGG. Specifically, this report observed a reduction in the metabolic pathways involved in purine, amino acids, pyrimidine, one-carbon metabolism, and secondary metabolites in LGG. This study proposes that this reduction plays a vital role in reducing the energy requirements and maintaining the carbon metabolism balance in LGG, thereby enabling it to survive and grow in cold conditions [34]. A model for the genes or central molecular pathways that are modulated by BLF supplementation in LGG, specifically when incubated in a cold environment, is explained in Figure 1 according to recent findings [34]. In this figure, we also added the roles of potential LF glycans and the transportation of LF into the cytosol of probiotic cells [35]. However, the prebiotic effects of lactoferrins on metabolic pathways in other probiotics remain poorly understood and warrant further investigation.

A recent review has summarized the influences of BLF or HLF on the growth of intestinal inhabitant bacteria, along with some simplified hypothetical mechanisms that may account for the in vitro growth-promoting effects of LFs on probiotics such as bifidobacteria and lactobacilli [35]. Specifically, it has been proposed that the prebiotic ability of LF is apparently independent of the BLF iron saturation extent, and the growth of probiotics may be facilitated through the binding of LF with probiotic proteins, which are then transported to the inner milieu for the concomitant cleavage and transport of LF-linked glycans used as energy sources. However, in our opinion, as discussed above, both our study and previous studies have reported that the prebiotic activities of HLF and BLF in vitro are dependent on the probiotic strains, and that BLF and HLF may even inhibit the growth of some probiotic strains. Therefore, we propose that the prebiotic mechanisms of BLF and HLF on specific probiotics are quite complex, and that the “simplified hypothetical mechanisms” of the prebiotic roles of LF [35] may only be applicable to the specific probiotic strains tested under the same conditions. Nonetheless, a recent study has developed a “the next generation of prebiotics” approach by covalent conjugation of a prebiotic indigestible carbohydrate to a peptic-hydrolysate of LF that provided resistance to gastric digestion to the peptide cargo. This dramatically improved the resistance of this cargo to gastrointestinal digestion, and the growth rate of a model probiotic bacterium (*L. casei*) on the conjugates was double that on the unconjugated components in an in vitro and simulated gastrointestinal digestion system [95]. In addition, the same group further developed a colonic delivery system selectively targeted to probiotic bacteria, made of oligosaccharides and LF hydrolysate conjugates, that self-assembled into core-shell particles. Importantly, the approach that has been proposed may provide a competitive edge to colon probiotics [96].

Collectively, further investigation is needed to fully understand the following issues: the molecular pathways of the prebiotic ability of LFs on most probiotic strains; the presence of LF-binding receptors (proteins) in all probiotic stains, especially given that the presence of LF-binding receptors on *Lactobacillus* is still unclear; and the interaction between commensal bacteria and the relatively low concentration of LF in most body sections. These could help to dissect the complex roles of LFs as prebiotic agents. Moreover, HLF and BLF are known to exhibit specific structural characteristics with respect to their iron-binding sites, glycan attachment sites, and glycan types. These characteristics have been recently reviewed and summarized by Vega-Bautista (2019) [35]. They are thought to reflect, in part, the multiple signaling pathways involved in the expression and regulation of the LF gene among mammalian species and gene polymorphisms. These features may also contribute to the prebiotic ability of LF, and further exploration is warranted. Nevertheless, we contend that LF administration (i.e., LF supplementation) in vivo provides more compelling evidence for promoting the growth environment of probiotics or improving the microbiota of the gut and reproductive tract, as discussed above.

## 6. Combining LF with Probiotics to Fight Bacterial Infections or Provide Beneficial Characteristics in Disease Control

Both probiotics and LF have been shown to have similar health-promoting abilities. Therefore, some studies have attempted to combine the potential benefits of LF with specific probiotics to fight against bacterial infections using different strategies. At first, Chen et al. found that BLF or BLF hydrolysate could not block the growth of most probiotic strains that were tested. Thus, they combined the supernatants of specific probiotics with BLF or BLF hydrolysate to test the antibacterial efficacy. This study discovered that the supernatants produced by *L. fermentum*, *B. lactis*, and *B. longum* blocked the growth of *E. coli* HER1255, *E. faecalis* ATCC 29212, *S.* Typhi, ATCC 19430, *S.* Typhimurium ATCC 13311, and *S.* Typhimurium ATCC 14028. Most significantly, a combination of apo-BLF or BLF hydrolysate with the supernatants of the above three probiotics showed synergistic or partially synergistic effects against the growth of most of the chosen pathogens [32]. Therefore, this demonstrated that several probiotic strains are resistant to apo-BLF and BLF hydrolysate, which warrants clinical studies to evaluate the antimicrobial potential of combining apo-BLF or its hydrolysate with specific probiotics. In addition, we found that BLF hydrolysate, but not complete BLF, inhibited the growth of most MRSA strains tested in vitro. Additionally, the supernatants produced by *L. fermentum* ATCC 11739, *Bifidobacterium longum* ATCC 15707, and *Bacillus lactis* BCRC 17394 inhibited the growth of various MRSA strains. When the supernatant of *L. fermentum* or *B. lactis* BCRC was combined with apo-BLF or BLF hydrolysate, it led to partially synergistic or synergistic growth-inhibitory activity against MRSA strains. Chen et al. also found that *L. fermentum*, but not *B. lactis* or *B. longum*, was resistant to the antibacterial activity of both apo-BLF and BLF hydrolysate. Therefore, these data support the idea that *L. fermentum* could be the best candidate to be used with apo-BLF or BLF hydrolysate as a live supplement against MRSA infections [97]. To support this, several clinical studies have reported that the addition of BLF and a specific probiotic formula to standard triple-eradication therapy could improve the *Helicobacter pylori* eradication rate and reduce the side effects in adults [98,99]. Furthermore, the combination of lactoferrin with probiotics has demonstrated the potential to reduce the incidence of necrotizing enterocolitis in preterm infants [63,100]. In addition, BLF supplementation, alone or in combination with LGG, is reported to decrease the risk of infections related to inhibitors of gastric acidity in very-low-birth-weight preterm infants [101].

Collectively, the above in vitro and clinical studies demonstrate the feasibility of combining specific probiotics with BLF or hydrolysate against bacterial pathogens or diseases in various fields. On the other hand, to facilitate the combination of LFs with specific probiotics, various studies, including ours, have attempted to construct recombinant probiotic strains that are capable of expressing HLF, BLF, or PLF. For instance, Yu et al. prepared four recombinant *lactobacillus* strains that expressed PLF, which were subsequently administered to mice for a duration of 14 days. A supplementation with PLF-expressing probiotics was found to increase both the average daily gain and average daily feed intake. Additionally, this supplementation exhibited a notable positive effect on the health of the animals against *E. coli* K88 or porcine pseudorabies virus infection in mice [102]. *L. casei* was chosen to deliver and express HLF, and an oral administration of this recombinant probiotic to mice was shown to help control gastrointestinal tract infections [103]. Similarly, Liao et al. developed a BLF-expressing *L. casei* strain and found that the recombinant *L. casei*/pPG612.1-BLF acted as an enhancer, improving the immunity of vaginal mucosa against the intrusion of *Candida albicans* in a murine model [104]. Moreover, *L. pentosus* was engineered to express PLF, and this recombinant PLF-expressing probiotic strain was demonstrated to increase the antibacterial activity in vitro and improve the efficacy of vaccination against Aujeszky’s disease in a mouse model [105].

Based on the above findings, previous studies have supported the feasibility of using probiotics to express various animal-derived LFs for clinical applications. However, there are still concerns among the public regarding genetically modified foods. Intriguingly, Liu et al. adopted different strategies, and they began to investigate the applicability of inactivated recombinant probiotics. For example, they have developed recombinant lactobacilli as promising producers of BLF, HLF, or PLF, and have further utilized the recombinant microorganisms by using disrupted probiotic lysates. As a result, this report obtained three recombinant LF-expressing probiotic strains, and their clear lysates significantly enhanced their antibacterial activities against important food-borne pathogens in vitro. Notably, these cell lysates did not carry transferable antibiotic resistance, which can be transferred to commensal or pathogenic bacteria [106]. This study suggests that these engineered probiotic strains provide both the beneficial characteristics of lactic acid bacteria and the biological activity of LF. To support this, this group further prepared three kinds of probiotic supplements from the above recombinant LF-expressing probiotics: lactic acid bacteria (LAB), LAB/LF, and inactivated LAB/LF. The LAB supplement was prepared from viable LAB without recombinant LF expression, the LAB/LF supplement was prepared from viable probiotics expressing LF, and the inactivated LAB/LF supplement was prepared from inactivated probiotics expressing LF. Furthermore, an oral administration of these probiotic supplements for four weeks significantly ameliorated diet-induced lipid accumulation and inflammation in nonalcoholic fatty liver disease (NAFLD) in a mouse model. Importantly, the data supported that the probiotics and LFs in the probiotic mixtures contributed differently to improving the efficacy against NAFLD, and the expressed LF content in probiotics could enhance their efficacy compared to the original probiotic mixtures. Moreover, when these LF-expressing probiotics were further inactivated using sonication, they showed a better efficacy against NAFLD than the viable probiotics [106]. Thus, this recent study has provided evidence supporting the potential of recombinant LF-expressing probiotics in improving hepatic steatosis.

Altogether, milk-derived BLF is currently easily obtainable, making its combined use with specific probiotic strains a favorable clinical strategy. Although previous studies have confirmed the ability of various functional LFs to be expressed through specific probiotics for clinical applications, our recent research has demonstrated that using inactivated recombinant strains with a clinical applicability is a more promising research direction due to the elimination of concerns regarding genetically modified foods.

## 7. Safety Issue

Lactoferrin (LF) and its related peptides are products that have been generally recognized as safe (GRAS) [107,108] and have been well tolerated in various studies [109,110,111] as well as in clinical trials [60,100]. For instance, BLF has been incorporated into infant formulas derived from bovine milk designed for infants aged 0 to 12 months, as well as toddler formulas for older infants and young children (13 to 36 months). The inclusion levels of BLF in these formulas can reach up to 100 mg/100 g of solid formula content. This concentration has been deemed acceptable by the Food and Drug Administration (FDA) as GRAS [107]. Additionally, BLF is naturally present in bovine milk, which has a long history of human consumption. It is a whey protein constituent of cow’s milk, comprising approximately 0.3% (0.1 g/L of milk) of the total milk protein content or 1.4% of the total whey protein content. Infants relying solely on cow’s milk-based formula, particularly those aged 0–5 months, have the highest estimated exposure to BLF, ranging from 75 to 137 mg/day. Moreover, studies investigating the acute oral toxicity of BLF have not identified any observed adverse effects, even at doses as high as 2000 mg/kg/day, as evidenced by both 4-week and 13-week sub-chronic toxicity experiments [107]. Furthermore, the estimated daily intake of LF for infants up to one year of age, which is approximately 210 mg/kg body weight, is about ten times lower than the highest dose (2000 mg/kg body weight per day) tested in a 13-week sub-chronic rat study, which did not exhibit any adverse effects related to BLF. For adults aged 19 and above, the proposed intake is approximately 100 times lower, and this level of anticipated intake is considered a high-intake scenario rather than a worst-case scenario. Consequently, this study concludes the absence of adverse effects from LF at the proposed consumption levels mentioned above [108].

Probiotics are also considered to be agents that are GRAS. However, there are concerns about the risk of probiotic-associated sepsis when administering live probiotics to immature infants [112,113]. Nonetheless, the absolute risk of sepsis from probiotic supplementation is likely to be low [114]. We have demonstrated that the use of inactivated or dead LF-expressing probiotics could help to control nonalcoholic fatty liver disease (NAFLD). We believe that using inactivated probiotics could be an alternative strategy in disease control [115,116].

## 8. Conclusions

In conclusion, LF or some peptides derived from its digestion seem to have a possible, but strain- and dose-dependent, prebiotic effect in vitro. Moreover, the molecular mechanism of LF to promote the growth of a specific probiotic strain has been dissected in part; but further studies are necessary to reach a conclusion. Nonetheless, the role of LF in improving gut dysbiosis or metabolic disorders is more convincing. Finally, the combination of LF with specific LF-resistant probiotics seems to be a promising strategy against bacterial infection or metabolic diseases. This could be achieved by directly combining the LF with specific probiotics or by using LF-expressing probiotics. Significantly, our study reveals that inactivated or dead LF-expressing probiotics have the potential to mitigate bacterial infections or NAFLD. This discovery presents an alternative way to apply LF in conjunction with probiotics.

## Figures and Tables

**Table 1:** Growth promotion or inhibition in probiotics cultured with bovine lactoferrin or its hydrolysate.

Probiotic	Form of Lactoferrin	Culture Condition	Dose and Key Features	References
*Bifidobacterium breve* BCRC 12584	Apo-BLF	22 °C, anaerobic	Dose–response growth (>1 mg/mL)	[33]
*Bifidobacterium longum* ATCC 15707	Bulk BLF	37 °C, anaerobic	A slight increase in growth response (2 and 4 mg/mL)	[29]
	Bulk BLF	37 °C, anaerobic	Growth was promoted (1 mg/mL)	[44]
*Bifidobacterium longum* ATCC 15708	Bulk BLF	37 °C, anaerobic	Good increase in growth response (2 and 4 mg/mL)	[29]
*Bifidobacterium longum* kd-5-6	Bulk BLF	37 °C, anaerobic	Good increase in growth response (2 and 4 mg/mL)	[29]
*Loigolactobacillus coryniformis* subsp. *coryniformis* ATCC 25602	Apo-BLF	22 °C, anaerobic	Dose–response growth (>1 mg/mL)	[33]
*Lactobacillus delbrueckii* BCRC 140	Apo-BLF	22 °C, anaerobic	Dose–response growth (>1 mg/mL)	[33]
*Lactobacillus acidophilus* BCRC 14065	Apo-BLF	22 °C, anaerobic	Dose–response growth (>1 mg/mL)	[33]
*Bifidobacterium angulatum* ATCC 27535	Apo-BLF	22 °C, anaerobic	Dose–response growth (>1 mg/mL)	[33]
*Bifidobacterium catenulatum* ATCC 27539	Apo-BLF	22 °C, anaerobic	Dose–response growth (>1 mg/mL)	[33]
*Lactiplantibacillus paraplantarum* ATCC 70021	Apo-BLF	22 °C, anaerobic	Dose–response growth (>1 mg/mL)	[33]
*Pediococcus pentosaceus* ATCC 8081	Apo-BLF	22 °C, anaerobic	Growth enhancement (>1 mg/mL)	[33]
*Lacticaseibacillus rhamnosus* ATCC 53103	Apo-BLF	22 °C, anaerobic	Growth enhancement (>1 mg/mL)	[33]
*Lacticaseibacillus paracasei* BCRC 17483	Apo-BLF	22 °C, anaerobic	Growth enhancement (>1 mg/mL)	[33]
*Lactobacillus acidophilus* ATCC 4356	Apo-BLF	37 °C, anaerobic	Growth inhibition 8 to 16 mg/mL	[32]
	BLF hydrolysate	37 °C, anaerobic	Growth inhibition 8 to 16 mg/mL	[32]
*Ligilactobacillus salivarius* ATCC 11741	Apo-BLF	37 °C, anaerobic	Growth inhibition 32 mg/mL	[32]
	BLF hydrolysate	37 °C, anaerobic	Growth inhibition 32 mg/mL	[32]
*Lacticaseibacillus rhamnosus* ATCC 53103	Apo-BLF	37 °C, anaerobic	Growth inhibition 1 to 16 mg/mL	[32]
	BLF hydrolysate	37 °C, anaerobic	Growth inhibition 1 to 16 mg/mL	[32]
*Bifidobacterium longum* ATCC 15707	Apo-BLF	37 °C, anaerobic	Growth inhibition 2 to 4 mg/mL	[32]
	BLF hydrolysate	37 °C, anaerobic	Growth inhibition 2 to 4 mg/mL	[32]
*Bifidobacterium lactis* BCRC 17394	Apo-BLF	37 °C, anaerobic	Growth inhibition 2 to 8 mg/mL	[32]
	BLF hydrolysate	37 °C, anaerobic	Growth inhibition 2 to 8 mg/mL	[32]
*Bifidobacterium bifidum* ATCC 15696	BLF hydrolysate	37 °C, anaerobic	Dose–response growth (0.01 to 1 mg/mL)	[45]
*Bifidobacterium longum* subsp. *infantis* ATCC 15697	BLF hydrolysate	37 °C, anaerobic	Dose–response growth (0.01 to 1 mg/mL)	[45]
	Apo-BLF	37 °C, aerobic, and anaerobic	Dose-dependent inhibition; MIC: 4 to 32 mg/mL	[46]
*Bifidobacterium breve* ATCC 15700	BLF hydrolysate	37 °C, anaerobic	Dose–response growth (0.01 to 1 mg/mL)	[45]
*Bifidobacterium bifidum* ATCC 29521	Apo-BLF	37 °C, aerobic, and anaerobic	Dose-dependent inhibition (>0.25 mg/mL); MIC: 128 mg/mL	[46]
*Limosilactobacillus reuteri* ATCC 23272	Apo-BLF	37 °C, aerobic, and anaerobic	Dose-dependent inhibition (>0.25 mg/mL); MIC: 64 to 128 mg/mL	[46]
*Loigolactobacillus coryniformis* subsp. *coryniformis* ATCC 25602	Apo-BLF	37 °C, aerobic, and anaerobic	Dose-dependent inhibition (>0.25 mg/mL); MIC: 4 to 8 mg/mL	[46]

## Data Availability

Not applicable.
